# Genomics: Encyclopedia of DNA

**DOI:** 10.1289/ehp.112-1277124

**Published:** 2004-08

**Authors:** Richard Dahl

The completion of the Human Genome Project in April 2003 was a landmark accomplishment, but much remains to be learned before scientists fully understand the true functionality of the DNA sequences in our genetic matter. To that end, the National Human Genome Research Institute (NHGRI) has initiated a variety of research projects to better understand the sequence. One of the most intriguing and potentially far-reaching of these efforts is the Encyclopedia of DNA Elements, or ENCODE, project, which aspires to create a complete catalog of all the functional elements of the human genome.

“The ultimate goal of the ENCODE project is to create a reference work that will help researchers fully utilize the human sequence to gain a deeper understanding of human biology, as well as to develop new strategies for preventing and treating disease,” says Elise A. Feingold, one of the NHGRI program directors in charge of the ENCODE project.

The goal of the Human Genome Project was simply to sequence the human genome; no distinctions were made between protein coding and noncoding regions. The ENCODE project is intended to pick up where the Human Genome Project left off, by providing answers about the roles that are played by the different genetic elements in the sequence. In addition to studying the human genome, the ENCODE project is also looking at genomic sequences from a variety of animals to provide multispecies comparisons. This will help to identify conserved sequences, which are thought to be strong indicators of functionally important regions in the human genome.

NHGRI launched ENCODE last year with the first round of a total $36 million in grants that will be awarded over a three-year period. The first round of awards went to 14 recipients in the United States and abroad. In addition to the grantees, several other academic and scientific groups are providing specific technical expertise, such as database coordination, to assist the project.

According to Feingold, the grantees and other contributors are working as a consortium to analyze about 1% of the genome. Their goal is to determine the most effective set of methodologies, which will then be applied to the remaining 99%.

One of the grantees is Anindya Dutta, a professor of biochemistry and molecular genetics at the University of Virginia. He and his colleagues are studying ways to map replication elements on human chromosomes. The completion of the Human Genome Project created what Dutta considers an obvious opportunity to embark on such a replication study.

“There are very few origins of replication mapped in human cells—five to ten if you’re generous, but I would say three or four,” Dutta says, referring to genetic elements that are necessary to initiate DNA synthesis. “It was pretty clear when the sequence of the human genome came out that this is a great tool for us to find hundreds of origins and how they are controlled by chromatin structure, gene density, promoter activity, and, of course, sequence.”

Following the model established by the Human Genome Project, NHGRI is calling for the data generated by the ENCODE project to be stored in databases and made freely available to the scientific community. The Center for Biomolecular Science and Engineering at the University of California, Santa Cruz—which is one of the institutions involved in providing support work for the ENCODE project and which also developed the computer programs that ran the sequencing of the human genome—is in charge of maintaining the database for sequence-related ENCODE data. In June 2004 the center added an ENCODE page (**http://genome.ucsc.edu/encode/**) to its existing genome browser, which gets 5,000 visits a day.

It’s not yet clear what might happen further with the data after the initial pilot project ends in 2006. Feingold says that when the first period ends, “we will evaluate what we have learned and determine the best path for moving forward.”

## Figures and Tables

**Figure:**
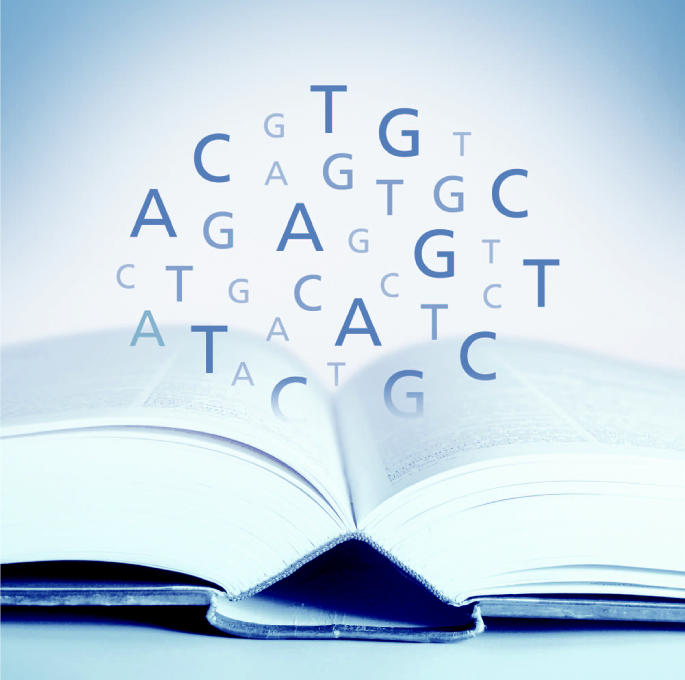
**Encyclopedia genomica.** The goal of the ENCODE project of the National Human Genome Research Institute is to create a complete catalog of all of the functional elements of the human genome.

